# Life and expectations post-kidney transplant: a qualitative analysis of patient responses

**DOI:** 10.1186/s12882-019-1368-0

**Published:** 2019-05-16

**Authors:** Emily L. Tucker, Abigail R. Smith, Mark S. Daskin, Hannah Schapiro, Sabrina M. Cottrell, Evelyn S. Gendron, Peg Hill-Callahan, Alan B. Leichtman, Robert M. Merion, Stephen J. Gill, Kayse Lee Maass

**Affiliations:** 1 Department of Industrial and Operations Engineering, University of Michigan, Ann Arbor, MI USA; 2 Arbor Research Collaborative for Health, Ann Arbor, MI USA; 3 Department of Internal Medicine, University of Michigan, Ann Arbor, MI USA; 4 Department of Surgery, University of Michigan, Ann Arbor, MI USA; 5 Stephen J. Gill Consulting, Ann Arbor, MI USA; 6 Department of Mechanical and Industrial Engineering, Northeastern University, Boston, MA USA

**Keywords:** Kidney transplantation, Grounded theory, Patient experience, Post-transplant expectations, Health-related quality of life, Uncertainty, Cost, Comorbidities, Qualitative research

## Abstract

**Background:**

The effect of a kidney transplant on a recipient extends beyond the restoration of kidney function. However, there is limited qualitative analysis of recipient perspectives on life following transplantation, particularly in the United States. To understand the full patient experience, it is necessary to understand recipient views on life adjustments after kidney transplantation, medical management, and quality of life. This could lead to improvements in recipient care and sense of well-being.

**Methods:**

We conducted a paper-based survey from March 23 to October 1, 2015 of 476 kidney transplant recipients at the University of Michigan Health System in Ann Arbor, Michigan. We analyzed their open-ended responses using qualitative research methods. This is a companion analysis to a previous quantitative report on the closed-ended responses to that survey.

**Results:**

Common themes relating to changes following transplantation included: improvements in quality of life, a return to normalcy, better health and more energy. Concerns included: duration of graft survival, fears about one day returning to dialysis or needing to undergo another kidney transplant, comorbidities, future quality of life, and the cost and quality of their healthcare. Many recipients were grateful for their transplant, but some were anxious about the burdens transplantation placed on their loved ones.

**Conclusions:**

While most recipients reported meaningful improvements in health and lifestyle after kidney transplantation, a minority of participants experienced declines in energy or health status. Worries about how long the transplant will function, future health, and cost and quality of healthcare are prevalent. Future research could study the effects of providing additional information, programs, and interventions following transplantation that target these concerns. This may better prepare and support kidney recipients and lead to improvements in the patient experience.

**Electronic supplementary material:**

The online version of this article (10.1186/s12882-019-1368-0) contains supplementary material, which is available to authorized users.

## Background

Kidney transplantation has transformed the care of patients with end-stage renal disease (ESRD). Each year, approximately 17,600 kidney transplants are performed within the United States (US), and currently 193,000 patients live with a functioning graft [1]. If the transplant functions for at least 1 year, the median survival of the kidney, depending on the donor type, is between 12 and 16 years [2]. Transplants can provide dramatic improvements in quality of life and health status [3]. However, improved outcomes are not universal [4], and most patients are expected to eventually suffer graft loss [2].

Despite the large and growing population of kidney transplant recipients, there is a limited understanding of the subjective patient experience following transplantation. A handful of qualitative studies have reported recipient perspectives. These include Boaz and Morgan, who interviewed kidney transplant recipients to study the return to normalcy, and Orr et al. who used focus groups to understand quality of life [4, 5]. They found that recipients define a normal life in a variety of ways and reported common themes of fear, gratitude, and a medicalization of their lives. While these teams spoke with 26 and 25 patients respectively, a wider sample may be better able to capture nuances of the post-transplant experience. Howell et al. used focus-nominal groups to study trade-offs between outcomes associated with immunosuppression [6]. They found that patients would rather experience severe treatment-related adverse events than lose their grafts, thereby illuminating the importance of understanding and including the patient perspective in clinical decision-making. Other studies in this area have used closed-ended questions to study patient experiences. Some compared life before and after transplant [7–9] and found that prior to transplant patients may overestimate gains in quality-of-life. One of the few studies that included prompts about the future was a follow-up of the Howell et al. qualitative analysis; they asked patients to rank post-transplant outcomes and found, supporting results from their earlier studies, that patients ranked graft loss worse than death [6, 10]. While there are reports on recipient perspectives from around the world (e.g., Europe [4, 5, 7]; Australia [6, 10]; Asia [11]; and Middle East [12]), there is little data on experiences and future expectations of recipients within the US. Experiences and interactions with healthcare systems may be strongly affected by the context of care, especially as it relates to cost and support systems.

This report is part of a larger study that seeks to understand the perspectives of kidney transplant recipients and their healthcare providers post-transplant. An earlier paper compared patient and provider responses to closed-ended survey questions from the same sample used in this analysis [13]. The objective of the current paper is to draw themes from open-ended responses about life changes, concerns about healthcare and future quality of life, and other reported post-transplant experiences.

## Methods

### Survey development

This study was approved by the University of Michigan (UM) Institutional Review Board (HUM00079279). The survey instrument was designed based on two initial focus groups (one with patients and one with providers). Each had five participants. Both groups lasted 1 h and included discussions about the post-transplant goals and expectations of kidney recipients. An experienced qualitative researcher (SJG) facilitated these sessions. After the survey was drafted, it was validated through two cognitive interview sessions to determine whether the participants understood the content of the questions. One session included three patient participants and the other five provider participants. The survey was revised based on this feedback. Further detail on the survey development and results from the closed-ended questions are presented in Maass et al. [13].

### Survey administration

The paper survey was administered between March 23 and October 1, 2015 to kidney transplant recipients and providers. The survey included both closed- and open-ended questions reflecting four main areas: provider-patient relationships; general and transplant-related health; elements of clinical care; and affect and well-being. The three open-ended response questions were: “How has your life changed since having a kidney transplant?”; “What concerns you most about your health care and future quality of life?” and “Is there something we didn’t ask you that you wish we had? If so, please tell us what it is.”

To participate in the study, patients were required to have had a primary kidney-only transplant at 18 years of age or older at UM; to be at least 1 month post-transplant; to have a currently functioning graft; and to not have had transplants of any other organ nor an additional kidney transplant prior to survey enrollment. Kidney transplants may have come from either living or deceased donors. Patients were recruited in-person during regular follow-up visits to the UM transplant center, a convenience sampling approach [14]. Each participant could read and understand English and provided written informed consent. Researchers were not present while participants completed the survey. Family members could have been in the room when the patient was consented and began the survey, and providers may have entered while participants were working on the survey.

### Data analysis

Demographics and transplant-related data were collected through retrospective chart review. Some demographic data from this study have been published elsewhere [13]. Reporting of race and ethnicity conformed to US federal guidelines [15, 16]. These data were used to compare participants who responded to any of the open-ended questions to those who left these questions blank. We adapted the COREQ guidelines to a qualitative survey analysis [17]. We report this alignment in Additional file 1.

#### Qualitative analysis

The analysis of the open-ended questions was conducted using methods from the grounded theory framework [18]. Themes of patient responses were developed through cascading analysis of responses, rather than through an a priori construct. In several steps, we iteratively reviewed patient responses and generated thematic categories based on the responses. After the final coding was completed, we combined categories into major themes.

Specifically, we first selected a random sample of 30 responses for each of the three questions. Three research staff members (ESG, HS, and SMC) each reviewed the responses for two of the three open-ended questions and developed initial response categories.

Next, the three research staff members reviewed all of the responses to the open-ended questions and identified additional response categories. The project team reviewed the revised list of categories, and the team combined overlapping categories to produce a preliminary list of ten, nine, and six categories for each question, respectively.

Using the preliminary lists of categories, all of the responses were coded (0 or 1) in Microsoft Excel to record whether they corresponded to each category, where 0 indicated no, and 1 indicated yes [19, 20]. Categories were not mutually exclusive; a response could correspond to multiple categories. Each question was coded by two independent reviewers (ESG, HS, SMC, or ELT), and inter-coder reliability was assessed at 94% or higher. During the coding process, new categories were proposed for each of the questions. After further team discussion, the new list of categories included 13, 11, and 13 categories for each question, respectively.

The responses to each of the questions were next re-coded according to the new list of categories. Each question was coded by two independent reviewers (HS, SMC, or ELT). No further categories were proposed, and the categorization was considered to have reached saturation [21, 22]. Initial discrepancies between coded responses were resolved through review by a researcher not involved with the coding (KLM). Where there was further uncertainty, discrepancies were resolved through consensus between two team members (KLM and ELT). Researchers (ESG, HS, SMC, and ELT) then identified representative quotations for each category for each question. Through a team discussion and review of the quotations, the categories were synthesized into a smaller number of overarching themes for a total of 6, 6, and 4 themes for each of the three questions, respectively. Participants did not provide feedback on the findings.

## Results

The response rate to the full survey was 66%, and demographic characteristics were similar between those who consented and those who declined to participate. Participants in the survey were older, transplanted more recently, more likely to be male and have received a living unrelated donor transplant compared to the UM kidney transplant population that met eligibility for the study. A comparison of the demographic characteristics of participants who responded to the open-ended questions to those who did not is shown in Table 1. Participants who answered the open-ended questions versus those who did not were younger at the time of transplant (mean age 48.3 vs. 52.0, *p* = 0.05), were more likely to be female (37% vs. 23%, *p* = 0.05), and were transplanted more recently (median years since transplant 5.1 vs. 8.0, *p* = 0.02).

**Table 1:** Survey participant demographics by free response status

	Sample with at least one free response (*n* = 428)	Sample with no free response data (*n* = 48)
Age at Transplant (mean [SD])	48.33 (13.57)	51.96 (11.75)
18–35 (% [n])	20% (84)	10% (5)
35–50 (% [n])	34% (148)	35% (17)
50–65 (% [n])	36% (154)	40% (19)
65–77 (% [n])	10% (42)	15% (7)
Gender % (n)		
Female	37% (160)	23% (11)
Male	63% (268)	77% (37)
Race % (n)		
White	77% (325)	79% (37)
Black	18% (76)	19% (9)
Asian	2% (10)	2% (1)
Other	3% (13)	0% (0)
Ethnicity % (n)		
Non-Hispanic	96% (390)	100% (47)
Hispanic	4% (15)	0% (0)
Donor Type % (n)		
Living Related	30% (128)	25% (12)
Living Unrelated	29% (126)	27% (13)
Deceased	41% (174)	48% (23)
Years Post-transplant on 10/1/2015 (median [IQR])	5.11 (2–9)	7.95 (3–11)
0.1–3 (% [n])	34% (144)	21% (10)
3–5 (% [n])	15% (65)	13% (6)
5–10 (% [n])	30% (128)	35% (17)
10–31 (% [n])	21% (91)	31% (15)

### Life changes post-transplant

In the first question, patients were asked: “How has your life changed since having a kidney transplant?” Six themes were identified from the 414 responses. Themes included improved quality of life and return to normalcy (*n* = 197); better health and more energy (*n* = 69); gratitude and sense of purpose or freedom (*n* = 55); burdens of post-transplant regimens (*n* = 37); no change (*n* = 30); and worsened and less energy (*n* = 23). These themes are reported in Table 2, and examples of responses are provided in Table 3. Thirty-nine patients wrote “yes” without further qualification. As it was unclear whether those changes were positive or negative, those 39 responses were not further analyzed.

**Table 2:** Themes from patient responses by question^a^

Question / Theme	Patient Responses (*N* = 476)^b^	Percentage among Respondents
Q13: How has your life changed since having a kidney transplant?	414	
Improved quality of life and return to normalcy	197	48%
Better health and more energy	69	17%
Gratitude and corresponding sense of purpose or freedom	55	13%
Yes	39	9%
Burdens of post-transplant regimens	37	9%
No change	30	7%
Worsened and less energy	23	6%
Q14: What concerns you most about your healthcare and future quality of life?	383	
Comorbidities and quality of life	162	42%
Kidney-related health issues	121	32%
Quality and cost of healthcare	84	22%
No concerns	40	10%
Family and support systems	24	6%
Lifestyle changes including less energy	22	6%
Q15: Is there something we didn’t ask you that you wish we had?	171	
No	115	67%
Post-transplant care	17	10%
More communication and information	11	6%
Medications	7	4%
Future of care and anxiety about health	5	3%

^a^Patients who did not express a particular theme did not necessarily disagree with the theme

^b^Patients were counted in each category they contributed responses to, so some patients are counted more than once

**Table 3:** Representative responses to the first open-ended question^a^

Patient Responses by Theme	
Improved quality of life and return to normalcy	
“Better by far then dialysis, I can have a daily schedule.”	
“More freedom/time (no dialysis), able to work and return to routine daily life.”	
“I can work now without missing much of it because of dialysis”	
“Back to work not as tired.”	
“I am able to do more things with my family”	
“I feel better. No more dialysis. Looking forward to doing things I couldn’t do.”	
“It has been much better. I am happy I got it.”	
“I now have a life. I can participate in activities that I couldn’t before. Even with many med I feel great.”	
“I am able to feel human and alive again”	
“Happy to not be hooked up to dialysis anymore almost to a point I forget I ever was.”	
“No more dialysis!! Wonderful!!”	
“Alive, alert, rested, peaceful, engaged in my tasks, much more positive outlook, paying more attention to overall health. I CAN SEE RETIREMENT!!”	
“Not doing dialysis 10 h per day, taking more meds, more free time.”	
Better health and more energy	
“I have been extremely healthy physically for 12 years now. So having health restored is an incredible experience. I appreciate life more.”	
“I feel awesome!!”	
“Overall better health + optimism”	
“I feel better all over. A feeling of well-being.”	
“More energy, clearer thinking, not sick daily, brighter outlook on life, happier”	
“I feel well the majority of the time”	
“More energy. Feel active. A normal active life.”	
“More energy + my outlook is more positive.”	
Gratitude and corresponding sense of purpose or freedom	
“I live life, enjoy even the small things, feel blessed.”	
“Every single day is a gift”	
“more freedom (dialysis)”	
“Freedom to travel. Being in control of my destiny.”	
“More freedom but also more health problem (diabetes) type 2”	
“Feel better and free.”	
“Being able to travel without the formality of finding a clinic to dialyze in while out of state”	
“more time (no dialysis), no sense of doom.”	
“It has been the most wonderful gift I have ever gotten. It has given me back my freedom to travel.”	
“more purposeful, more appreciation.”	
“It has become full of more purpose. I feel I have been given a 2nd chance.”	
“More energy + my outlook is more positive.”	
“due to meds I am hospitalized 3–5 times a year. Moody. Able to travel some. Thankful for each day, life is short.”	
Burdens of post-transplant regimens	
“Lots of meds”	
“Just taking more pills”	
“Quality of life is little changed, but it’s a hassle to take meds every 12 h.”	
“Not eating what I want continuously taking meds; feeling tired; afraid of losing my kidney.”	
“The only change has been the medication regimen. Over the term to date of my transplant I have taken more than 85,000 pills”	
Worsened and less energy	
“No pep, tired”	
“Have slowed down, not as much energy. Sometimes just don’t care”	
“More stressful”	
“Radical Changes, Unable to work due to secondary problems from ESRD & Diabetes”	
“Yes, other conditions worse.”	
No Change	
“No”	
“It has not changed”	
“Not much”	
“Very little”	

^a^The first question was “how has your life changed since having a kidney transplant?” Responses have been lightly edited to correct spelling errors

#### Improved quality of life and return to normalcy

The most common change noted after a kidney transplant was an improvement in quality of life and a return to normalcy. For many, no longer attending dialysis clinic multiple days per week allowed them to, as one patient wrote, “have a daily schedule” and as another patient wrote, have a “routine daily life.” Some reported that they have the time post-transplant to work, spend time with their families, and conduct tasks of daily living. Many patients also could use this time in new ways because they felt better, describing it as “I now have a life. I can participate in activities that I couldn’t before.” This led to a strong sense of normalcy for some; e.g., “I am able to feel human and alive again” which may in turn have caused the stress of their pre-transplant lives to fade. As one patient wrote: “happy to not be hooked up to dialysis anymore almost to a point I forget I ever was.”

The enthusiasm expressed in the survey responses varied, but many conveyed strong emotion. This was expressed in intense punctuation, “No more dialysis!! Wonderful!!” and phrases written in all capital letters: “I CAN SEE RETIREMENT!!” Even in more muted responses, e.g., “It has been much better. I am happy I got it”, the change was evident. In writing about the extremes of pre-transplant life, some patients may have used hyperbole to show a dramatic contrast with their post-transplant lives, e.g., “not doing dialysis 10 hours per day.”

#### Better health and more energy

Many patients reported improvements in their health. Some of these responses were particularly strong; e.g., “I feel awesome!!” and “having health restored is an incredible experience.” However, some of the subdued responses also affirmed the substantial improvement that can occur after a transplant; e.g., “overall better health” and “I feel better all over.” One patient reported “not sick daily,” and another wrote “feel well the majority of the time.” Many also commented on increased energy.

#### Gratitude and sense of purpose and freedom

Recipients discussed the relief they felt from the burdens of renal failure and dialysis. One patient wrote “enjoy even the small things, feel blessed,” and another reported “every single day is a gift.” Many participants specifically mentioned feeling an increased sense of freedom, e.g., “feel better and free.” This was often presented in the context of no longer having regular dialysis visits, e.g., “able to travel without the formality of finding a clinic to dialyze in while out of state” and “no sense of doom.” Another patient reported that “it [the transplant] has been the most wonderful gift I have ever gotten. It has given me back my freedom to travel.” Some mentioned a new sense of conviction. One felt “more purposeful,” and another reported, “it [their life] has become full of more purpose. I feel I have been given a 2^nd^ chance.” A recipient reported “being in control of my destiny.” Patients also noted a greater sense of hopefulness; one reported “optimism,” another commented “more positive,” and a third wrote they are “thankful for each day, life is short.”

#### Burdens of post-transplant regimens

Patients also reported undesirable changes to their lives after transplantation. Often these were related to medications. Recipients take medications at least twice daily to prevent rejection and to treat comorbidities. Some patients were matter-of-fact, e.g., “lots of meds” and “just taking more pills.” Others alluded to the burden of the required medication; e.g., “it’s a hassle to take meds every 12 hours” and “continuously taking meds.” For some, changes in medication defined their post-transplant experience. A recipient wrote that “The only change has been the medication regimen. Over the term to date of my transplant I have taken more than 85,000 pills.” Others mentioned the dietary restrictions that can be added after a transplant, including as one wrote “not eating what I want.”

#### No change

Some patients reported that they did not experience substantial changes after receiving a transplant. Many of these responses were simply “no.” Another recipient wrote “it has not changed,” and others reported “very little” or “not much.”

#### Worsened and less energy

Finally, some patients reported having less energy, describing it as “no pep” and “have slowed down… sometimes just don’t care.” They also reported instances of poor transplant outcomes, many of which were related to comorbidities. One patient wrote “radical changes” and that he or she is “unable to work due to secondary problems from ESRD & Diabetes.” Another reported “other conditions worse.”

### Concerns about their healthcare and future quality of life

The second open-ended question asked: “What concerns you most about your health care and future quality of life?” Three hundred eighty-three patients responded, and six themes were identified: kidney-related health issues (*n =* 121); comorbidities and quality of life (*n* = 162); quality and cost of healthcare (*n =* 84); no concerns (*n =* 40); family and support systems (*n =* 24); and lifestyle changes including less energy (*n =* 22). These themes are reported in Table 2, and examples of patient responses are provided in Table 4.

**Table 4:** Representative responses to the second open-ended question^a^

Patient Responses by Theme	
Kidney-related health issues	
“concerned about kidney failure”	
“Rejection or failure of the transplant.”	
“Possible kidney rejection”	
“One day I know my transplant will fail”	
“fear of future unknown, how long will my kidney last rejection”	
“How long will kidney last”	
“How long will transplanted kidney last and what will happen to health then.”	
“Having to go on dialysis once this kidney loses function”	
“Never want dialysis again”	
“That I will be able to function and feel good when I am 20+ years older. Also, that I will be able to receive another transplant when needed.”	
Comorbidities and quality of life	
“diabetes”	
“Blood Pressure & Diabetes”	
“That my Lupus will flare up and cause me to decline or kill my kidney.”	
“Feet pain due to neuropathy”	
“Cancer & Heart Disease”	
“Skin cancer due to transplant”	
“Multiple skin cancers”	
“That I’m going to die with cancer in my stomach”	
“repeat UTIs”	
“Heart disease - wish I had more energy.”	
“pain, numbness, balance, and coldness”	
“Function on my own and continue to be healthy”	
“stay healthy”	
“continue to live an active high quality life”	
“to be able to do the things I did before. Do stuff with my grandchildren and friends. Hang out laundry outside, go where I want to go”	
“Staying healthy and not being a burden to others. Being able to do what I want without pain.”	
“diabetes, fatigue, not being able to work-I never know when I will go back in the hospital (frustrating)”	
Quality and cost of healthcare	
“COST!!!!”	
“How much all of it will cost?!”	
“the cost”	
“Insurance, insurance, insurance. Extremely important that I get excellent insurance to cover all my meds to the end of my life, which will be over 100 years young.”	
“Rejection of insurance”	
“Losing my health insurance.”	
“Insurance and its possible disruption to the best healthcare.”	
“I just want the future level of care to continue as it is at present”	
“My concerns are the cost of health care, what quality of health care will be provided in the future, what will my options be choosing concerning the health care, how will my health care be different in 10 to 20 years”	
Family and support systems	
“I do not wish to become a burden on my family”	
“My children are grown now (22 yrs. & 20 yrs). It has been 7 yrs. since my transplant and I’m sure they would like to move out & live a normal life but feel quite guilty trying to help take care of me & help with all the bills.”	
“I know my transplanted kidney will not last forever. So I’m most concerned about getting sick again and going through dialysis and another surgery and the impacts it will have on my family now that I have a child.”	
“Being taken care of”	
Lifestyle changes including less energy	
“Being mobile and able to take care of myself.”	
“Getting to walk again.”	
“Heart disease - wish I had more energy.”	
“How will my finances be managed in the future if my transplant kidney fails and I cannot work as [a] mechanic?”	
“I worry that I will be able to work until I am at retirement age”	
“I hope not to have issues in the future. My schooling and career are important for me to finish/figure out.”	

^a^The second question was: “what concerns you most about your healthcare and future quality of life?” Responses have been lightly edited to correct spelling errors

#### Kidney-related health issues

Patients expressed concerns about kidney failure and how long their transplants will last. Several responses were variants of “failure of the transplant.” However, for some, the concern was not *if* graft loss would occur but rather *when*. One reported the “fear of future unknown, how long will my kidney last,” and another wrote “not knowing how long my kidney will perform and if my health will deteriorate again.” Patients also expressed concerns about what would happen after their transplant failed. Many commented specifically on returning to dialysis, including “having to go on dialysis once this kidney loses function”, and “never want dialysis again.” Others were concerned about a second transplant, e.g., “be able to receive another transplant when needed.”

#### Comorbidities and quality of life

A predominant concern was for comorbidities related to kidney disease and quality of life. Patients often noted a particular comorbidity that they either currently have or could develop in the future. Many answered solely “diabetes.” Other conditions mentioned included high blood pressure, pain, cancer, recurrent urinary tract infections, and heart disease. Patients also described symptoms, e.g., “pain, numbness, balance, and coldness.”

A large portion of participants reported improvements in their quality of life in the first open-ended question, and many used this space to express concerns about maintaining it. Several responses were close variants of “staying healthy,” and one recipient wrote he or she wanted to “continue to live an active high quality life.” Another responded: “to be able to do the things I did before. Do stuff with my grandchildren and friends. Hang out laundry outside, go where I want to go.” Another included the concern of “being able to do what I want without pain.” Others expressed concern about the risk of hospitalization, e.g., one reported “never know when I will go back in the hospital (frustrating).”

#### Quality and cost of healthcare

Some of the strongest responses were related to the affordability of post-transplant care. The exclamation of one patient – “COST!!!!” – may reflect the strain that transplant-related financial difficulties can place on patients and their families. Frequent responses within this theme were short variants of “the cost.” Another patient wondered rhetorically: “How much all of it will cost?!” Financing, including insurance, was another worry; e.g., “insurance, insurance, insurance.” Responses included “rejection of insurance,” “losing my health insurance,” and “insurance and its possible disruption to the best healthcare.”

Others were concerned about the quality of their healthcare. One noted “I just want the future level of care to continue as it is at present,” and another recognized potential changes in the long-term: “how will my health care be different in 10 to 20 years.”

#### Family and support system

Participants also commented on the significant impacts a transplant can have on family structure and roles. These responses were often imbued with emotion, and some expressed concern about how their future care would affect their families; e.g., “I know my transplanted kidney will not last forever. So I’m most concerned about getting sick again and going through dialysis and another surgery and the impacts it will have on my family now that I have a child.” A few mentioned the possibility of becoming a burden; e.g., “I do not wish to become a burden on my family,” and another said, “I’m sure they [his/her grown children] would like to move out & have a normal life but feel quite guilty trying to help take care of me & help with all the bills.” One reported the concern “being taken care of.” Other patients were concerned about the opposite role - being able to support their families, e.g., “not being there for my family” and “not being able to take care of my family.”

#### Lifestyle changes including less energy

Patients also mentioned concerns related to lifestyle changes. Some noted the desire to be more mobile, e.g., “getting to walk again” or have more energy, such as “wish I had more energy.” Others were concerned with their ability to work; one wondered “How will my finances be managed in the future if my transplant kidney fails and I cannot work as [a] mechanic?”

### Additional topics

The final open-ended question asked: “Is there something we didn’t ask you that you wish we had? If so, please tell us what it is.” One hundred seventy-one patients responded. Most respondents wrote “no” (*n =* 115), indicating that they did not have anything to add. Of the patients who included additional comments, four themes were identified: post-transplant care (*n =* 17); more communication and information (*n =* 11); medications (*n =* 7); and future of care and anxiety about their health (*n =* 5). These themes are reported in Table 2, and additional examples of responses are provided in Table 5.

**Table 5:** Representative responses to the third open-ended question^a^

Patient Responses by Theme	
Post-transplant care	
“You have answered all the questions and I felt comfortable, life has been a blessing and my kidney doctors have done a great job. Keep up the good work.”	
“How is the scheduling, wait times, value for wait times (i.e. is it worth waiting for 1.5 h to see someone from 10 mins and then the travel times etc.) Perception of true health care or is it more of a billing opportunity”	
More communication and information	
“Didn’t explain all the after effects in results of transplant. Weight gain, eating too much (prednisone) heart problems maybe, etc.”	
“There is often no concern at the hospital (when hospitalized) about my poor immune system and right dosage of my medication. (and not listening to my correction and causing invalid blood count.)”	
Medications	
“Being able to afford my meds”	
Future of care and anxiety about health	
“Having a transplant introduces some insecurity in that future health is uncertain as if it is very dependent on graft survival and graft function. I have been extremely fortunate in maintaining my graft for over 17 years- which I consider a near miracle.”	

^a^ The third open-ended question was: “is there something we didn’t ask you that you wish we had?” Responses have been lightly edited to correct spelling errors

Many of these themes overlap with previous questions or report an individual recipient’s concern with their own care. Notably, some wished for more information from their providers and more communication. One patient wrote: “didn’t explain all the after effects in results of transplant,” and another reported “there is often no concern at the hospital… about my poor immune system and right dosage of my medication (and not listening to my correction and causing invalid blood count).”

## Discussion

This analysis provides a broad view of the patient experience following kidney transplantation. Questions prompted recipients to consider how their lives had changed and their concerns about their healthcare and quality of life. Many themes emerged from the responses, including experiencing higher quality of life and better health since transplantation as well as concerns about future health, costs, and burdens of care. These themes echo and extend results from studies of kidney transplant recipients in other contexts [3–6, 8, 10, 11].

Our results suggest that patients have disparate experiences post-transplant. When asked “How has your life changed since having a kidney transplant?”, the largest portion of patients reported improvements in quality of life and health, while several reported that there had been no change, and some said their lives had worsened. This is consistent with Orr et al. ([4] citing Orr A: “Patient attitudes to renal transplantation and quality of life following renal transplantation: an exploratory study,” unpublished)— who found that 15% of transplant patients experienced difficulties—and other studies that have reported improvements [3, 24]. Similarly, prior studies have reported different results for changes in energy level; some have seen increases [3, 8], and others have reported decreases [5, 11, 23]. Patient responses in this study lend credence to the finding that energy levels after kidney transplantation vary between recipients. Similarly, participants reported a variety of common comorbidities including diabetes, cancer, and heart disease [6], though these do not affect all patients.

In addition, while many patients expressed the desire for a normal life, patients may have different views on what a “normal life” is. Some recipients mentioned that they have returned to work. Others reported that they were better able to care for themselves and others, travel, and spend time with their families. While providers may consider returning to work an important metric for transplant success [23, 25–27], the range of responses indicate that patients may define normalcy more broadly [5].

The effects of a kidney transplant are not limited to a patient’s physical health or lifestyle; it can also have substantial psychological benefits. Patients expressed feeling a heightened sense of purpose and gratitude to be alive. For many, this manifested itself in an increased sense of freedom. While Smith et al. did not find statistically significant differences in the amount patients traveled pre- vs. post-transplant [8], recipients can travel without having to make cumbersome dialysis arrangements to maintain their dialysis regimes while away from their customary dialysis facilities. The optimism may also be derived from how much better many patients feel. As their health improves, it is perhaps natural to foresee the trajectory of their lives improving as well.

However, some patients also expressed a psychological weight from changes post-transplant. A large portion reported concerns regarding the potential for graft failure. Past studies that have asked patients to rank possible transplant outcomes found that patients considered graft failure worse than death [6, 10] and that the risk of graft loss is a persistent fear [5]. Our study adds to past literature in that it expands on *why* graft failure is concerning. Patients reported stress related to the uncertainty about when the failure would occur and the implications for life afterwards. The anxiety about returning to dialysis may be due to both the physical toll and the potential burden on their support systems. Furthermore, some patients expressed an obligation to live a life of purpose because of the gift of the transplant; this echoes a prior study that reported some patients felt a duty to care for their kidney [4].

We see a resonance in many of the patient perspectives with other studies conducted with patients outside of the US [4–6, 10, 11]. This lends support to the sense that the themes of quality of life and health post-transplant and views on graft failure and living a life of purpose may be experienced broadly.

In contrast, other prominent concerns included cost and insurance, which may be more important within the US context. These were among the strongest responses, perhaps because they threaten both quality of life and health. In particular, some of these concerns may relate to the US payment system for immunosuppressant medications. After a kidney transplant, Medicare pays for the immunosuppressant medications for the first 3 years but not after that time (unless the patient qualifies for Medicare via age or disability). Medications that are not covered by insurance can cost thousands of dollars out of pocket annually [28, 29]. Patients also expressed specific concerns about insurance. This may affect how patients evaluate the decision to return to work; e.g., patients may be unlikely to work part-time if it would lead to a loss of government benefits. In the time since the survey was conducted (2015), public policy in the US regarding healthcare funding and insurance availability for those with preexisting conditions has been volatile. This may further exacerbate feelings of insecurity related to costs.

Our study is among the few to use qualitative methods to study kidney transplant recipients within the US context and to examine expectations for the future. Much of the qualitative research about life after a kidney transplant has been conducted through focus groups and interviews, in contrast to in-person surveys. Our study sample was recruited during follow-up visits and included a broad range of patient demographics and times since transplant; it is possible that a wider range of patients participated than those who would have attended more time-intensive focus groups.

The study’s limitations should also be considered. In contrast to studies that use interviews and focus groups, the survey was not interactive. As such, we could not ask patients for clarification or for additional detail. We therefore must be particularly careful with interpretation and be aware of a negativity bias. The open-ended questions were asked at the end of the survey; responses may have been influenced by earlier survey questions or survey fatigue. Not all participants responded to the three open-ended questions; 87, 80, and 36% of the participants answered the questions, respectively. The study was conducted at a single center, and it should be noted that the exclusion criteria barred children and patients with non-renal or prior renal transplants from participation, so responses may not fully generalize to the full spectrum of kidney transplant recipients. Patient responses were collected in 2015 and may be less applicable in today’s context. Perspectives, particularly those related to insurance and the cost of post-transplant care, may also not be representative of patient concerns outside of the US. Finally, the counts presented in Table 2 should be viewed cautiously. A lack of response does not imply that the patient agreed or did not agree with a theme.

## Conclusions

In this follow-on study, we use qualitative methods to analyze the perspectives of kidney transplant recipients about their current experiences and future concerns. Common themes in the responses included improvements in quality of life and health, as well as concerns about remaining healthy, future quality of life, and cost. Further study may be helpful to understand the effects of cost on patient care and experiences. The findings also suggest the need to study whether changes in education and practice could lessen the stress related to uncertainty in transplant outcomes. A better understanding of recipient perceptions based upon insights gained from qualitative studies of recipient experiences may lead to improvements in pre-transplant education and in post-transplant care.

## Additional files

Additional file 1: COREQ Guidelines Checklist. This file documents the study’s correspondence to the COREQ guidelines. (DOCX 30 kb)

Additional file 2: Open-Ended Responses. This file includes the participant responses to the three open-ended questions. (XLSX 45 kb)

## Abbreviations

ESRD: End-stage renal disease
UM: University of Michigan
US: United States
