# Supplementary material for: Life and expectations post-kidney transplant: a qualitative analysis of patient responses
Source: BMC Nephrol. 2019 May 16;20:175. doi: 10.1186/s12882-019-1368-0 (PMC6524208; doi:10.1186/s12882-019-1368-0)
Supplement: Supplementary file 1 — COREQ Guidelines Checklist. This file documents the study’s correspondence to the COREQ guidelines. (DOCX 30 kb) [file 12882_2019_1368_MOESM1_ESM.docx]

COREQ Guidelines Checklist

In this file, we report how the analysis in Tucker et al. aligns with the COREQ guidelines [1]. The guidelines are intended to be used for focus groups and interviews, and we identify items that do not apply to open-ended survey responses as not applicable.

**Domain 1**: **Research Team and Reflexivity**

These items are not applicable for surveys.

**Domain 2: Study Design**

*Methodological orientation and theory:* We applied elements from the grounded theory framework [2].

*Sampling:* convenience sampling approach

*Method of approach:* Study coordinators approached kidney transplant recipients, who were waiting to be brought to a clinic room for a routine follow-up appointment, to participate in a written survey. Open-ended free response questions were included as part of this survey.

*Sample size:* 476 eligible patients took the written survey; 428 patients responded to at least one free response question

*Non-participation:*

- 230 patients declined to take the survey (reasons not given)
- 24 patients were found to be ineligible after the survey given
  - 20 patients did not receive a transplant at the University of Michigan
  - 4 patients received transplants when they were under 18 years old
- 48 patients did not respond to any of the free response questions

*Setting of data collection:* Clinic rooms at the University of Michigan Transplant Center outpatient transplant follow-up clinics

*Presence of non-participants:* Family members and healthcare providers may have been present. Research staff members were not present when the surveys were taken.

*Description of sample:* The survey was conducted between March 23 and October 1, 2015. The demographic characteristics were presented in an earlier publication [3] as well as Table 1 of this manuscript.

*Interview guide:* The questions were provided by the authors. The research team conducted two interview sessions with three patients and five providers, respectively, to validate the questions.

*Data saturation:* We considered the categories to have reached saturation. This is discussed in further detail in the “qualitative analysis” subsection of the Methods section.

The following items are not applicable to surveys:

- Repeat interviews
- Audio/visual recording
- Field notes
- Duration
- Transcripts returned

**Domain 3: Analysis and Findings**

*Number of data coders:* Four researchers coded the responses. One researcher reviewed discrepancies.

*Description of the coding tree:* The coding process is described in the text in the “qualitative analysis” subsection of the Methods section. In the following tables (A-1; A-2; and A-3), we present the categories the researchers used to code the responses for each question and the corresponding overarching themes.

***Table A-1:*** *Categories and themes for question 13: “How has your life changed since having a kidney transplant?”*

| **Categories (n=13)** | **Themes (n=6)** |
| --- | --- |
| No change | No change |
| Yes | Yes^§^ |
| No dialysis | Improved quality of life and return to normalcy |
| Good, better quality of life, or normalcy |  |
| Better health | Better health and more energy |
| More energy or more active |  |
| Purposeful or appreciation | Gratitude and corresponding sense of purpose or freedom |
| Freedom |  |
| Negative eating habits | Burdens of post-transplant regimens |
| Medications |  |
| Worsened | Worsened and less energy |
| Less energy |  |
| Positive eating habits | *Removed* |

^§^ Response counts reported but not considered a theme.

***Table A-2:*** *Categories and themes for question 14: “What concerns you most about your healthcare and future quality of life?”*

| **Categories (n=11)** | **Themes (n=6)** |
| --- | --- |
| Comorbidities and quality of life | Comorbidities and quality of life |
| Kidney failure and how long kidney will last | Kidney-related health issues |
| Dialysis |  |
| Cost of care | Quality and cost of healthcare |
| Medications |  |
| Quality of health care remaining |  |
| No concerns | No concerns |
| Family and support systems | Family and support systems |
| Ability to work or go to school | Lifestyle changes including less energy |
| Mobility |  |
| Less energy |  |

***Table A-3:*** *Categories and themes for question 14: “Is there something we didn't ask you that you wish we had?”*

| **Categories (n=13)** | **Themes (n=4)** |
| --- | --- |
| No | No^§^ |
| Satisfaction (good) with post-transplant care | Post-transplant care |
| Satisfaction (bad) with post-transplant care |  |
| Wished for more communication | More communication and information |
| More information |  |
| Meeting with other recipients |  |
| Medications, including cost | Medications |
| Future of medicine | Future of care and anxiety about health |
| Impact on family or spouse |  |
| Insecurity |  |
| Weight control | *Responses re-coded into other themes* |
| Transplant donors | *Removed* |
| Future of health |  |

^§^ Response counts reported but not considered a theme.

*Derivation of themes:* Derived from the data

*Software:* Microsoft Excel [4]

*Participant checking*: The respondents were not asked for feedback on the results.

*Quotations presented:* In the manuscript, representative quotations of the responses are provided in the Results section and Tables 3, 4, and 5. In Additional File 2, we report all of the responses, sorted by a de-identified study number.

*Data and findings consistent:* The findings are consistent with the data. Readers can verify the findings with the raw data provided in Additional File 2.

*Clarity of major themes:* All themes are presented in the Results section and in Tables 3, 4, and 5 in the manuscript.

*Clarity of minor themes:* All themes are presented in the Results section and in Tables 3, 4, and 5 in the manuscript.

**References**

1. Tong A, Sainsbury P, Craig J. Consolidated criteria for reporting qualitative research (COREQ): a 32- item checklist for interviews and focus group. Int J Qual Heal Care. 2007;19:349–57.

2. Barney G. Glaser and Anselm L. Strauss. The discovery of grounded theory; strategies for qualitative research. Chicago: Aldine Pub. Co.; 1967.

3. Maass KL, Smith AR, Tucker EL, Schapiro H, Cottrell SM, Gendron E, et al. Comparison of patient and provider goals, expectations, and experiences following kidney transplantation. Patient Educ Couns. 2018. doi:10.1016/j.pec.2018.12.010.

4. Microsoft. Excel. 2016. https://www.microsoft.com/en-us/download/details.aspx?id=49130.
